# Civil Society Involvement in Managing the COVID-19 Pandemic: A Case Study of a Large and Congested Urban Community in Bangkok, Thailand

**DOI:** 10.1089/heq.2023.0215

**Published:** 2024-04-15

**Authors:** Suwatjana Noinam, Noppawan Piaseu, Tiraporn Junda, Sukanya Tantiprasoplap, Phachongchit Kraithaworn, Suphanna Krongthaeo, Jantra Keawpugdee, Wanna Sanongdej, Saowaros Kongcheep, Wasana Srisuk

**Affiliations:** ^1^ Ramathibodi School of Nursing, Faculty of Medicine, Ramathibodi Hospital, Mahidol University, Bangkok, Thailand.; ^2^ Center of Health Promotion and Well-Being, Faculty of Medicine, Ramathibodi Hospital, Mahidol University, Bangkok, Thailand.

**Keywords:** COVID-19 pandemic, urban community, civil society, community management, Thailand

## Abstract

**Introduction:**

The COVID-19 pandemic has significantly impacted Thailand, including urban centers like Bangkok and surrounding areas, highlighting a critical need for effective management within densely populated communities to mitigate its effects.

**Methods:**

This qualitative study sought to explore community management strategies developed in Khlong Toei, a large, congested urban community in Bangkok, Thailand. Seven in-depth interviews (*n*=7), six focus groups (*n*=23), and two brainstorming sessions (*n*=12) were conducted for this study. Data were collected using Zoom, an online communication platform, and through on-site interviews between August 2021 and March 2022 in the congested urban community of Bangkok, Thailand. The data were analyzed using content analysis.

**Results:**

All informants (age range: 20–66 years, female respondents: 73.33%) were recruited by a community leader and the abbot of Saphan Temple, the community waiting area in Khlong Toei. The findings revealed two main themes: (1) Caring people, including two subthemes, and (2) Caring community, including two subthemes.

**Discussion:**

The study's findings provide guiding inputs for management of public fear to prevent emerging or re-emerging infectious pandemics within congested urban communities.

## Introduction

Given the rapid spread of the COVID-19 disease worldwide and its potential for increased severity and prevalence, the World Health Organization declared it a global pandemic.^1^ In Thailand, the pandemic was characterized by three distinct phases. The first two phases emerged from January to March 2021, with limited and controlled spread in specific areas. The third and largest wave initiated in central Bangkok in April 2021.^2^ Consequently, the pandemic spread throughout the country, particularly in densely populated areas. Congested urban communities like Khlong Toei, which accommodates over 100,000 people, are especially vulnerable to widespread human-to-human transmission of the virus,^3^ given that large families often live together in small houses. As of October 1, 2022, Bangkok had the highest number of infections in the country, with a total of 970,118 confirmed cases.^4^

COVID-19 has significantly decelerated the global economy, placing millions of jobs at risk and jeopardizing the food security and nutritional status of countless people. It has also disrupted daily life,^5–7^ particularly in low-income countries.^8^ The disease has widely affected public health, causing illness and death, with common symptoms being fever, flu, cough, pain, breathing difficulties, and potentially pneumonia.^5^ In Thailand, a sharp increase in new infections and more severe symptoms were evident during the third COVID-19 wave. This situation has posed a strain on health care resources, resulting in shortages of hospital beds and medical equipment and inadequate personnel to care for patients. In addition, uncoordinated patient referral and inadequate follow-up information systems have contributed to the high death rate.^3^

Furthermore, the spread of COVID-19 has affected Thai families, especially those living in Khlong Toei communities. They were highly vulnerable to COVID-19 due to their unstable economic condition, limited access to social welfare, poor housing, overcrowded neighborhoods, and polluted environment.^9^

Khlong Toei, one of Bangkok's 50 districts, is a large urban community with diverse housing, including multistory buildings and closely spaced houses. Despite its limited space, the area accommodates hundreds of thousands of residents within ∼12 km^2^. Some people reside in unregistered residences, facing challenges such as limited access to health care, especially for migrant workers and those relocated from other provinces. Government efforts for COVID-19 screenings and medical care encounter challenges due to a shortage of health care personnel, resulting in disparities in public service access.

The majority of Khlong Toei's population experienced economic hardships, making them vulnerable to the pandemic. Despite public rapid screening initiatives, accessing fair health care remained challenging, with a shortage of health care professionals and an inadequate system failing to serve the economically disadvantaged. Limited space for isolation posed difficulties, with some people waiting at home for available hospital beds. In addition, mild or inconclusive symptoms may leave some unaware of infection. The community expressed alarm and fear about the COVID-19 pandemic. The Thai government's lockdown and social isolation strategy in 2020–2021 significantly impacted residents in densely populated urban communities, where social distancing was particularly challenging.^9^

Amid a strained public health system struggling with a surge in infections, Saphan Temple's monks in the Khlong Toei district, a respected local institution, had recognized the community's distress. Collaborating with leaders and Nongovernmental Organizations (NGOs), they actively sought solutions, such as transforming the temple into a temporary shelter for patients awaiting hospital admission, aiming to enhance the community's crisis management. This initiative emphasized the community's effective management and resilience, guided by the belief that “by working together, we can navigate through the crisis collectively.” This study aimed to explore community management strategies for controlling the COVID-19 pandemic in Khlong Toei. It provided an approach for managing public fear and preventing emerging or re-emerging infectious pandemics in communities facing crises of similar contexts elsewhere.

## Methods

### Research design

This qualitative study is part of a larger research project titled “Impacts and Community Resilience in the COVID-19 Pandemic: A Synthesis.” The data were obtained through brainstorming, secondary sources, nonparticipant observations, in-depth interviews, and focus group discussions (FGDs), guided by semistructured interview questions. The data were analyzed using content analysis.

### Research setting

This study focused on one specific community within the Bangkok metropolitan area, Thailand. In this community, certain COVID-19 management strategies were carried out, which can serve as examples for future improved epidemic management strategies. Covering a total area of 12,316 km^2^, it comprises 3 subdistricts, with a total of 71,692 households and 101,892 persons, excluding the unregistered population.^10^ Due to the densely populated nature of the community, there was limited space for isolation. The hospitals lacked sufficient beds to accommodate patients. As the head monk appointed by the National Office of Buddhism, the Abbot, along with monks and community leaders collaborated with local NGOs to effectively manage the crisis. Their collaborative efforts successfully decreased the impact of the pandemic and alleviated the community's concerns.

### Research instrument

The semistructured interview questions used for brainstorming, in-depth interviews, and FGDs were open-ended, focusing on activities related to community management during the COVID-19 pandemic. Examples of these questions include: How did you or your family cope with COVID-19? Please describe your responsibilities in managing COVID-19 care. What are your primary concerns and anxieties while providing care for COVID-19, and how do you address them? Within your community/organization, how have issues related to the pandemic been handled and resolved? What challenges were encountered, and what recommendations do you have for improving and strengthening response efforts to effectively mitigate the impact of the COVID-19 outbreak? Additional questions relevant to the study's objectives were later incorporated to gain further insight. Multiple data sources were utilized, and researcher meetings were organized for triangulation. Data were verified with the original informants to ensure the quality of the obtained data.

### Ethical considerations

This study was approved by the Human Research Ethics Committee of the Faculty of Medicine, Ramathibodi Hospital, Mahidol University, Bangkok (approval No. COA.MURA 2021/706). The researchers contacted potential informants telephonically to inquire about their willingness to participate in the project. They introduced themselves, explained the research objectives, data collection methods, risks, and benefits. They also informed informants of their rights to withdraw from the research at any time without facing negative consequences and to communicate with project managers. Key informants provided informed consent before participating in this study. The researchers prioritized their privacy and honored their decisions, ensuring the protection of the rights and privacy of the informants. The study results were evaluated based on ethical principles, including (1) respect for person, (2) beneficence or nonmaleficence, and (3) justice.

### Data collection

The researchers contacted key informants through gatekeepers, the community leader, and the abbot who provided researchers with contact information and consequently researcher could contact potential participants, utilizing a purposive and snowball sampling method. There were a total of 30 key participants, categorized into 6 groups: (1) Monks including 4 participants, consisting of 1 abbot and 3 monks, (2) Community leaders including 4 individuals, (3) Public sector officers consisted of 6 individuals, including the director of the Society and Health Institute, Ministry of Public Health, a representative from the Port Authority of Thailand, a physician, a nurse from the public health center, and 1 professional nurses from private and public hospitals, (4) NGOs/volunteers included 10 participants, consisting of representatives from 3 civil groups and 5 volunteers who were former patients, (5) 5 former patients, and 6) a journalist.

They were selected to be the representatives of people who play major role in managing pandemic crisis to limit the spread of COVID-19 pandemic in the Khlong Toei community.

This qualitative study used the following data collection methodologies: brainstorming, in-depth interviews, nonparticipant observation, secondary data, and FGD. The researchers conducted seven in-depth interviews, six FGDs, and two brainstorming sessions. The data collection was conducted on Zoom, an online communication platform and on-site interviews were conducted between August 2021 and March 2022. The questions used in the in-depth interviews, group discussions, and brainstorming sessions were reviewed by three experts: a nurse instructor, a psychiatrist, and a community medicine specialist. These experts provided suggestions and modifications to ensure the validity of the questions for data collection.

Following approval of the study by the Human Ethics Committee, the data collection process started with an initial 60 min brainstorming session, aimed at comprehending overall situations and issues relevant to participants such as monks, community leaders, public sector officers, civil groups/volunteers, former patients, and a journalist, totaling 12 informants. Subsequently, in-depth interviews were conducted to gain a comprehensive understanding of experiences and management strategies, involving five former patients with COVID-19, the abbot, and a responsible nurse. FGDs were then carried out to explore management strategies, including community leaders, Buddhist monks, community volunteers, nurses and physicians, as well as representatives from civil societies and journalists. A final brainstorming session was conducted via the “Zoom” or online platform. On-site meetings were arranged if informants expressed discomfort with online communication.

The approach used depended on the convenience and availability of each participant. The researchers followed the in-depth interview and focus group guidelines when asking questions, and informants were able to provide information or express their opinions independently. Each session lasted between 60 and 120 min, and video and audio recordings were made. If any information was incomplete or unclear, follow-up clarification interviews were conducted until no new information emerged, indicating saturation of data.

### Data analysis

The data were analyzed using content analysis, involving verbatim transcription, reading the transcripts, and analyzing them by assigning codes and grouping-related content. Code samples from the transcripts were identified to highlight similarities and differences. The data were identified to reflect on the activities related to the process of COVID-19 management. Data on the same issue were derived from multiple sources, including in-depth interviews, FGDs, brainstorming, and nonparticipant observations. The researchers used the triangulation technique using multiple data collection methods such as brainstorming, secondary sources, nonparticipatory observations, in-depth interviews, and FGDs.

The research team analyzed the data, subsequently confirming its accuracy by cross-verifying it with information obtained from the interviews and rechecking with key informants for clarification. The analysis was conducted concurrently with data collection, and all data were subsequently reviewed with the informants for triangulation purposes. To ensure trustworthiness, credibility, dependability, transferability, and conformability were considered in this study. The researchers verified and analyzed the acquired data with the research team to minimize potential bias and confirm the research results.

## Results

The findings presented evidence gathered from monks, community leaders, public sector officers, NGOs, former patients or relatives, and journalists. Table 1 shows the characteristics of the 30 key informants. Two recurrent themes were found in the analysis namely: (1) Caring people consisting of two subthemes: Providing isolation units for sick people and Reducing fear, and (2) Caring community consisting of two subthemes: Providing food to stop leaving home and Creating volunteers in the community. Each of these themes is discussed below.

**Table 1.** The Demographic Characteristics of Informants (*n*=30)

Demography	***n***	%
Age (in years)		
Adult (20–59)	25	83.33
Older adult (60–66)	5	16.67
Gender		
Male	8	26.67
Female	22	73.33
Religion		
Buddhist	29	96.67
Muslim	1	3.33
Marital status		
Single	18	60.00
Married	10	33.33
Divorced/widowed/separated	2	6.67
Educational attainment		
No formal education up to elementary education	5	16.67
Secondary education up to a bachelor's degree	17	56.67
A graduate degree	8	26.66
Role within community		
Public sector officer	6	20.00
Monk	4	13.33
Community leader	4	13.33
Former patient or their relative	5	16.67
Government organization	1	3.33
Journalist	1	3.33

### Caring people

This involves caring for people in crisis, causing disruptions in the community. If patients remain at home, there is a risk of spreading the infection to close family members. As a result, some individuals are forced to sleep in their cars, while certain communities have established temporary isolation areas using space in community health centers and large transparent plastic sheets to temporarily separate infected individuals awaiting transfer to the hospital. However, this is insufficient given the increasing number of patients each day. In addition, there is community resistance due to fears that it may become a hotspot for the disease. The abbot of Saphan Temple is deeply troubled by this situation. He sees these people as his relatives, those who once offered alms to him. He wonders how he can assist them. This involves two subthemes: providing isolation units for sick people and reducing fear.

### Providing isolation units for sick people

As the daily count of new COVID-19 infections rose, patients faced challenges in accessing hospitals due to a shortage of beds, and in Khlong Toei, most households lacked dedicated isolation spaces. Concerns about isolation grew within the community members. The community initiated the establishment of a waiting center in response to this situation. The abbot of Saphan Temple proposed using the temple space as a makeshift field hospital, establishing a “Waiting Center” to serve as a comprehensive interim care facility for patients with COVID-19. In addition, it plays a crucial role as a landmark for ambulance pick-ups, facilitating the transportation of patients to the hospital.

Collaborating with community leaders and civil society, the monks played a significant role in transforming a nine-story building at Saphan Temple into a temporary center for patients awaiting transfers. The first floor served as a zone for admitting new patients, the second floor accommodated the residence of Buddhist monks, and the third to eighth floors provided housing for patients, with separate areas for men and women. Initially, there were 200 beds, later expanded to 500 beds. This is the sole center in the Khlong Toei area, serving as a prototype and the first of its kind in Bangkok.

Many informants noted that …

We didn't get a higher education like some others, but we believed in ensuring the survival of our community. That's why we've gathered our community members here to send to the hospital. In some homes, there were as few as six people, while in ours, there were 13 people in separate rooms, but they all shared the same exit door. We thought that if we stayed together, we might die together. (A community leader, female, 59 years old)

We've outlined the approach, [namely] that if someone got infected, they should came out [of their home] and waited at a temporary center at Saphan Temple. If they are were able to leave for a transfer [to the hospital], they [will] do so; if not, they [will] remain in waiting. (An abbot, male, 66 years old)

Originally, we only accepted cases from the Khlong Toei area. However, because of the insufficient number of beds in hospitals, we extended our assistance beyond our boundaries. We did not discriminate based on nationality or religion. We view all [people] as human beings and provide help to one another. (A monk, male, 49 years old)

### Reducing fear

The abbot of Saphan Temple not only provided accommodation but also ensured care to alleviate the fear among the community members. This care encompassed both physical and mental well-being, including monitoring the symptoms of illness. Moreover, there was an emphasis on open communication about the realities of COVID-19, fostering confidence, reducing stigma, and enhancing community unity. The two subthemes include monitoring symptoms and improving whole-health and public communication regarding up-to-date COVID-19 information.

### Monitoring symptoms and improving whole health

The Saphan Temple was not just a place for patients awaiting transfers but the Buddhist monks also help care infected people in this. The original intention behind establishing the isolation center at Saphan Temple was to provide temporary accommodation for infected people with COVID-19 awaiting hospital transfers. The goal was to avoid overnight stays at the temple. However, the shortage of beds in hospitals meant that patients had to remain at the center for over 24 h. This shelter did not have regular health care personnel; rather, consultations are organized using the LINE application and phone calls. The Buddhist monks served as a temporary substitute, providing dedicated care around the clock at the facility to the best of their ability. Within the building, there are closed-circuit cameras installed to monitor the condition of patients.

Monks and volunteers observe the patients' condition through the monitor screen, and designated volunteers on each floor provide regular care. In cases where a patient's condition appears unstable, the volunteer team conducts assessments every 2 h. Furthermore, Junior Buddhist monks and volunteer assessors evaluated the patients' condition using the LINE application. If any abnormal symptoms are observed, the information is forwarded to relevant organizations for coordination and further assistance. The Buddhist monks remain steadfast in providing moral support, genuinely caring for patients and delivering food, medicine, or requested items. After the abbot observed a deterioration in the condition of untreated patients, COVID-19 patients were given permission to use herbal remedies, including andrographolide and ginger, oral medication, and herbal steaming and baths, with the aim of alleviating the initial COVID-19 symptoms. The Buddhist monks acted as a temporary offering dedicated care. Some informants experienced that …

Moreover, the abbot assessed and cared for patients, providing medications based on their symptoms. If someone has a cough, they're given cough medicine. If there's a fever, they're provided with fever-reducing medicine. Additionally, remedies like andrographolide, ginger juice, or fingerroot are available. In most cases, medication is distributed according to the specific symptoms. (Monks, male, 49 years old)

When asked about their worries, they (patients) often mention fear. In such cases, I would suggest using the Dhamma (Buddhist teachings) to alleviate their concerns. Each monk had a different psychological approach, but they all try to help patients to reduce their anxiety. I, for instance, spoke through a microphone every morning, delivering Dhamma teachings for them to listen to. (A monk, male, 49 years old)

The patient refused to eat because he could not taste anything due to a loss of taste. He didn't want to consume food as it lacked flavor. The monk advised him, ‘Don't eat for pleasure; we eat to nourish our bodies, to fuel ourselves in the fight against the illness. If you don't eat, you won't have the strength to combat the disease for sure. Therefore, you must eat to gain the strength to fight it. Alongside taking medication, it will improve.’ He believes in this approach. (A monk, male, 49 years old)

### Public communication regarding up-to-date COVID-19 information

There has been an increase in providing information about COVID-19 to reduce fear within the community, diminish stigmatization, and dispel misconceptions about those infected. This communication involves sharing experiences directly from patients to the general public through face-to-face conversations. Community leaders also engage in public relations using community radio to disseminate information throughout the community. In cases where patients have completed the 14-day treatment and isolation period, the temple arranges for private vehicles to conduct chest X-rays at the temple. In addition, coordination with the Khlong Toei district is undertaken to obtain a certificate confirming the completion of the isolation period, allowing them to return into the community. Some informants experienced that …

In the initial stages, when recovered patients returned home, there was apprehension in the community about potential infection transmission. It was crucial to communicate to the community that those who had undergone treatment and recovered were no longer capable of spreading the virus. This information, disseminated through news channels and social media, helped alleviate community fears. Initially, there was some aversion and reluctance towards these patients returning, but through public relations efforts and explanations, understanding improved. Otherwise, recovered patients would face difficulties returning home. (A community leader, female, 55 years old)

Actually, this disease is not as terrifying as one might think. And for those who have recovered well, it's important to continue sharing knowledge because there may be new strains in the future. Emphasis is placed on providing information to take care of oneself, ensuring quick recovery and preventing unnecessary anxiety. (A community leader, male 37 years old)

We continue to treat based on symptoms until they complete the 14 days. We also issue a complete 14-day isolation certificate. For those who need an X-ray, they register, and when it's time, they come for the X-ray appointment. (A monk, male, 49 years old)

### Caring community

This is a community care initiative to encourage compliance with the country's policies to reduce the spread of COVID-19 on a broader scale. The COVID-19 pandemic led the Thai government to declare a state of emergency across the nation. The government promoted the public health campaign “stay at home, stop the virus, for the nation” in an effort to flatten the epidemic curve. This campaign urged people to stay at home as much as possible and only leave for essential needs, such as purchasing food, groceries, or seeking medical attention. Moreover, the severe and urgent lack of resources led to the natural emergence of community volunteers for the survival of the community. There were two subthemes relating to the informants' perceptions, providing food to stop leaving home and creating volunteers in the community, and adjusting living habits; a new way of life.

### Providing food to stop leaving home

While patients in quarantine at the community center received food support, there is a possibility that some patients and at-risk individuals isolated at home lack family readiness to handle their food needs. The Khlong Toei community did not hesitate to respond to this issue. Community leaders had come together to setup up a community kitchen, where meals are prepared using donated ingredients. This effort is intended to keep patients and at-risk groups confined within their homes. Almost all community members rely on the food provided, as many have been affected by work stoppages and income loss. One participant stated that …

Many of the volunteer cooks were older women who are not employed and who worked as housekeepers. They came together to help. On certain occasions, we prepared up to 1,000 boxes of food, as the community consists not only of those infected but also people who have been furloughed or impacted by COVID-19. There are unemployed because of the pandemic, and we provided meals for them as well. We provide three full meals delivered to their doorstep, with another committee member acting as the delivery person. Additionally, we receive donated boxed meals, which alleviate the burden of preparing all three meals, as our kitchen has been consistently providing meals for the past five months. (A community leader, female, 55 years old)

### Creating volunteers in the community

During the COVID-19 crisis in the Khlong Toei community, volunteerism has emerged in the community, including various ages and roles, with the goal of collaboratively caring for people affected by the impacts of COVID-19. This included establishing kitchens to distribute meals to patients and high-risk groups in quarantine. In addition, those who had recovered from COVID-19 volunteered to care for patients within the community. Some also volunteered to drive and transport patients from the community to quarantine centers or hospitals. They also care for high-risk people under home quarantine. Some of these volunteers previously contracted COVID-19 and wish to draw on their experiences to help others. Some informants experienced that …

We need synergy in the community, whether it's the volunteer spirit or committees. We had to come together because a few people [working alone] can't manage the work effectively. There were always two sides; for instance, the cooks prepared the food, and the volunteers delivered the meals. (A community leader, female, 55 years old)

I was afraid at first, but if I don't take action, who will the community rely on? If the community leader is still fearful, how can the members find support? I consider myself a pillar and need to coordinate with various organizations to disseminate information to the community members. I drew strength from the abbot who advised us to take medication according to symptoms. It's not as simple as it seems. So, I adopted the abbot's mindset to take care of myself and others. (A community leader, female, 59 years old)

What we observed is the emergence of new leaders in the community, such as in the Khlong Toei market area. There is a merchant in the market who transformed into a leader responsible for overseeing and coordinating assistance to undocumented migrant workers. This merchant, along with many others, assists in financial matters and coordinates transportation to the hospital for them. (A public sector officer, male, 45 years old)

## Discussion

The key findings of this study were (1) caring people and (2) caring community. These findings reflected efforts to break the transmission chain and alleviate panic within the community. The immediate activities emphasized separating infected patients from their families. Anyone with a COVID-19 infection, whether or not they are symptomatic, can transmit the virus to others through actions such as coughing, sneezing, talking, or prolonged close contact.^11,12^ Furthermore, people who are infected or at high risk in this community do not have separate spaces for isolation because of cramped living conditions and a lack of space. Some families share living spaces and utilities, making disease prevention challenging. This situation resembles countries such as India, where resources are limited. Those living in large families within small homes or in cramped slums find it practically impossible to follow the guidelines for isolating to reduce the risk of household transmission.^13^

The concept of isolation for infected people may seem simple for those whose homes have multiple rooms. Monks, community leaders, and NGOs in Khlong Toei took immediate action by implementing measures to break the chain of transmission, particularly by separating infected patients from their families. They worked rapidly to identify and reach these patients, aiming to minimize the spread of COVID-19 within the densely populated community. In response to the absence of separate spaces for isolation, they established a waiting center within the community. This strategy aligns with approaches seen in countries like China, where rapid containment measures, including isolating virus-positive groups, identifying at-risk groups, conducting testing, and quarantining close contacts, were simultaneously implemented within communities. The comprehensive strategy aims to mitigate the risk of infection spread.^14^

The Khlong Toei community took the initiative to create a waiting center due to the unavailability of hospital beds. They selected the historic Saphan Temple as the site for a community isolation center for COVID-19 patients, with the aim of breaking the chain of transmission. Saphan Temple was chosen due to its longstanding proximity to the community. The establishment of the Saphan isolation center resulted from collaborative planning and efforts involving the community, the temple, government, and nongovernment organizations. In collaboration with community leaders, Buddhist monks from Saphan Temple converted the Dhamma school building at the temple into a waiting center for infected patients who could not be treated at home (Home Isolation).

The center was sustained through public donations and supplies, exemplifying the community's resilience in confronting the challenges posed by COVID-19. This initiative has served as an exemplary for other communities. Subsequently, Thailand's Ministry of Public Health initiated a policy of establishing community isolation centers to minimize internal movement and reduce the spread of the disease.^15^ Home Isolation was established as a place for those with mild symptoms to isolate themselves due to the shortage of health personnel and insufficient beds for COVID patients in hospitals.

The Saphan isolation center marked Thailand's inaugural and singular establishment, where Buddhist monks proactively and systematically cared for patients throughout their illness until recovery. It served as an exemplary for other communities to emulate. During periods of patient numbers surpassing the health care system's capacity, Buddhist monks voluntarily intervened to prevent a situation where community members, awaiting medical care at home faced delays due to long queues. In Cambodia, Khmer monks not only played crucial roles in Buddhism but also took on significant roles in political situations and crises in Khmer cities, serving as healers, practitioners, and modern psychiatrists.^16^ Similarly, during a disaster in Sri Lanka, Buddhist monks established temporary welfare centers in temples for displaced people. They provided disaster victims with physical, mental, and spiritual support and coordinated various actors in disaster mitigation and resettlement projects.^17^

In times of crisis, collaboration, compassion, and the active involvement of individuals become paramount. The temple in Khlong Toei had long been an anchor for the community, and some Buddhist monks were descendants of this very community, remaining steadfast in their service. The Buddhist monks lived with the people's happiness, sadness, and suffering, believing that their well-being was intertwined with the community's.

Due to the novel and easily transmissible nature of COVID-19, coupled with the potential for fatalities, the public is significantly alarmed and fearful. The monks at Saphan Temple in Khlong Toei actively work to alleviate fear among patients. They use herbal remedies to address symptoms, closely monitor patients through monitors to assure them that they are not alone and utilize Buddhist teachings to bring calmness to patients' minds. In addition, public communication enhances the community's understanding of the disease, reducing stigma, fear, and mutual suspicion. Previous studies have shown that people often lack a clear understanding of the transmission and nature of the COVID-19.^18,19^

Some experience extreme fear and discomfort when thinking about or encountering information related to the pandemic.^20^ Vulnerable groups, such as family members of infected patients and residents of high-incidence areas, face social rejection and discrimination.^21,22^ Therefore, primary care and accurate information communication regarding COVID-19 are crucial to managing uncertainties and fears.

Working alongside the monks, the new generation of community volunteers tends to members' various needs, ranging from illnesses to hunger, thus showcasing the strong collaborative ties between the temple and the community helped the Khlong Toei community to navigate the COVID-19 crisis. This collaborative effort, much like the initiatives of Iran's communities, during the initial outbreak, personal protective equipment was scarce and difficult to procure. Therefore, volunteers joined forces to produce masks and gloves, secure essential items for health care, provide online learning tools for unprepared children, purchase food for infected families, and offer psychological support. Moreover, they cooperated with governmental agencies to screen at-risk groups.^23^

In the Khlong Toei community, central kitchens have been established to prepare and distribute meals to those infected as well as those impacted by job losses, thereby reducing the need for movement. During the periods of lockdown, all workplaces, educational institutions, business centers, and transportation were partially or fully closed to prevent the COVID-19 transmission and everyone was encouraged to stay at home. Consequently, many people lost their jobs and income, particularly day laborers, small business owners, and those from low-income households.^24–27^ Dealing with the disease alone, without considering people's lives and existence, is insufficient.

The spread of COVID-19 involves the dynamic nature of people, who are constantly on the move, particularly in densely populated communities with insufficient savings to remain idle without work. The fear of starvation outweighs the fear of COVID-19. If the community is assured of having enough food to sustain life, they can cease movement and comply with lockdown measures. Similar challenges have been observed in the United States, where the pandemic has reduced incomes and created job instability and food insecurity. Households facing food insecurity are less likely to adhere to social distancing guidelines, which puts them at higher risk, with few means of avoiding this risk.^28^ Securing food is equally imperative and cannot be overlooked. If community members are confident that they have sufficient food for their sustenance, they can remain in place without needing to travel.

The community of densely populated Khlong Toei District was among the first in Thailand to be proactive in tackling the COVID-19 situation. The community-based isolation center at Saphan Temple serves as an essential self-management model within the community and the temple by fostering a self-care network linked to the more extensive governmental system. This initiative reflects the power of community collaboration, which drove successful COVID-19 initiatives. Community leaders, Buddhist monks, and volunteers within robust local networks ensured the community's survival.

Due to the outbreak of COVID-19, an emerging infectious disease, we have learned about management strategies. Preparedness involves the following key elements: (1) Isolation units equipped with sufficient facilities and meeting established standards, (2) Clear and prompt communication of accurate information regarding the pandemic situation and management guidelines to alleviate public fear, and (3) Effective leadership involves establishing networks, engaging and managing community members serving as volunteers, and continuously training them for the knowledge needed in responding to and managing urgent situations.

## Abbreviations Used

FGD: focus group discussion
NGO: nongovernmental organization
